# Inflammatory response: The target for treating hyperpigmentation during the repair of a burn wound

**DOI:** 10.3389/fimmu.2023.1009137

**Published:** 2023-02-01

**Authors:** Chi Zhong, Geao Liang, Peiting Li, Ke Shi, Fuyin Li, Jianda Zhou, Dan Xu

**Affiliations:** Department of Plastic Surgery, The Third Xiangya Hospital, Central South University, Changsha, China

**Keywords:** inflammatory response, hyperpigmentation, burn wound, treatment, cytokines, postinflammatory hyperpigmentation

## Abstract

Hyperpigmentation is a common complication in patients with burn injuries during wound healing; however, the mechanisms underlying its occurrence and development remain unclear. Recently, postinflammatory hyperpigmentation (PIH) was found to result from overproduction of melanin. Local or systemic inflammatory responses are often observed in patients who develop hyperpigmentation. However, we lack studies on the relationship between PIH and burn injury. Therefore, we comprehensively reviewed the existing literature on the melanogenesis of the skin, inflammatory mechanisms in pigmentation, and local or systemic alteration in inflammatory cytokines in patients suffering from burn trauma to elucidate the relationship between PIH and burn injury. We believe that this review will guide further research on regulating melanin production in the burn management process.

## 1 Introduction

An estimated 11 million burn injury cases are reported annually worldwide (1). Approximately 50%–60% of the affected people, especially those with a dark complexion, develop hyperpigmentation at the burn injury site (2). Hyperpigmentation is a common condition that increases the economic burden of patients and reduces their quality of life by leaving an irreversible dermatological abrasion and severely affecting their psychological health. Postinflammatory hyperpigmentation (PIH) is a reactive and acquired hyperpigmentation of the epidermis and dermis, with variable pathogeneses and etiologies, including dermatoses, burn injury, and cosmetic procedures (3–5). Among these, burn trauma, especially severe burns, should never be ignored, as it is associated with pigmentation disorders (6, 7) as well as increased systemic and local inflammatory activities (8, 9). However, further studies are required to establish the relationship between PIH and burn injury-related inflammatory responses. Therefore, we have presented an overview of melanogenesis, mechanisms of PIH, and inflammation induced by burn injury. We have discussed their potential relationships, the limitations of the present research, and an orientation for future research.

## 2 Melanogenesis

Skin phenotype in individuals is mediated by the deposition of melanin granules in keratinocytes. These granules are transported from epidermal melanocytes through melanosomes (10, 11). Each melanocyte can interact with 40 viable keratinocytes adjacent to its dendrites, forming an epidermal melanin unit (12). The mature melanosomes carrying melanin granules are transferred from the dendrites of melanocytes into the cytoplasm of keratinocytes through exocytosis, cytophagocytosis, plasma membrane fusion, and membrane vesicle transfer (11, 13–15). The movement of microtubules, actin cytoskeleton, centrosomes, and centriolar satellites in keratinocytes carries the melanin-laden melanosomes to the supranuclear region to form microparasols, thereby protecting the epidermal DNA from UV-induced stimuli or damage (16). Conversely, keratinocytes, fibroblasts, and immune cells regulate pigmentation through hormones and cytokines (17).

Neural crest cells, migrating from the dorsolateral and ventral route, differentiate into melanoblasts by wingless-related integration site (WNT) signaling and subsequently form melanocytes in the hair follicles and epidermis (18, 19). Melanosomes, the tissue-specific lysosome-related organelles (LROs) located in the melanocytes, are the factories synthesizing and packing melanin.

**Figure 1:**
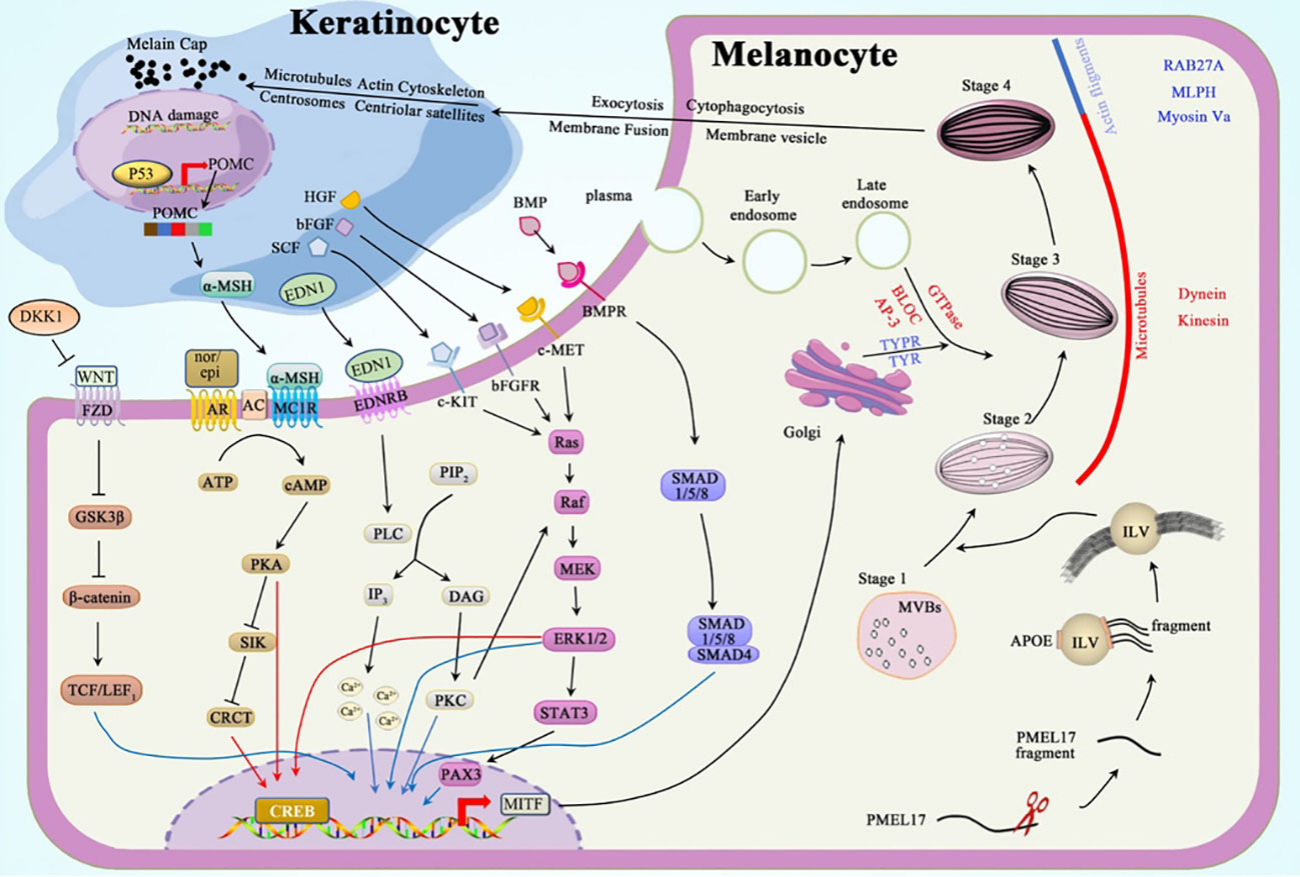
Melanogenesis and regulation.

Melanosomes undergo maturation through four stages (**Figure 1**): Stages I–II include premelanosomes, which cannot synthesize melanin until they mature to stages III–IV. Stage I melanosomes originate as multivesicular bodies (MVBs) containing intraluminal vesicles (ILVs) and melanocyte protein (PMEL17) (20). PMEL17 fragments are cleaved from the pre-melanosomal membrane and bound to the ILV surface. This process is modulated by apolipoprotein E (ApoE); this explains the elliptical shape of eumelanosomes since stage II, whereas pheomelanosomes are spherical owing to the suppression of PMEL17 expression by agouti signaling (21). Concordantly, the shape of melanosomes depends entirely on the form of PMEL17 fragments, and the reduced levels of these protein fibrils lead to the morphological disruption of melanosomes (22, 23). In stage III, melanogenic enzymes, tyrosinase (TYR) and tyrosinase-related protein-1 (TYRP1), are transported from the Golgi apparatus into melanosomes to produce melanin to be deposited on the amyloid fibrils of PMEL17 (24). In stage IV, the melanin accumulation on these fibrils is complete, contributing to the heavy pigmentation of melanosomes observed under the electron microscope (EM) (25).

### 2.1 Melanin biogenesis and transport within melanosomes

**Figure 2:**
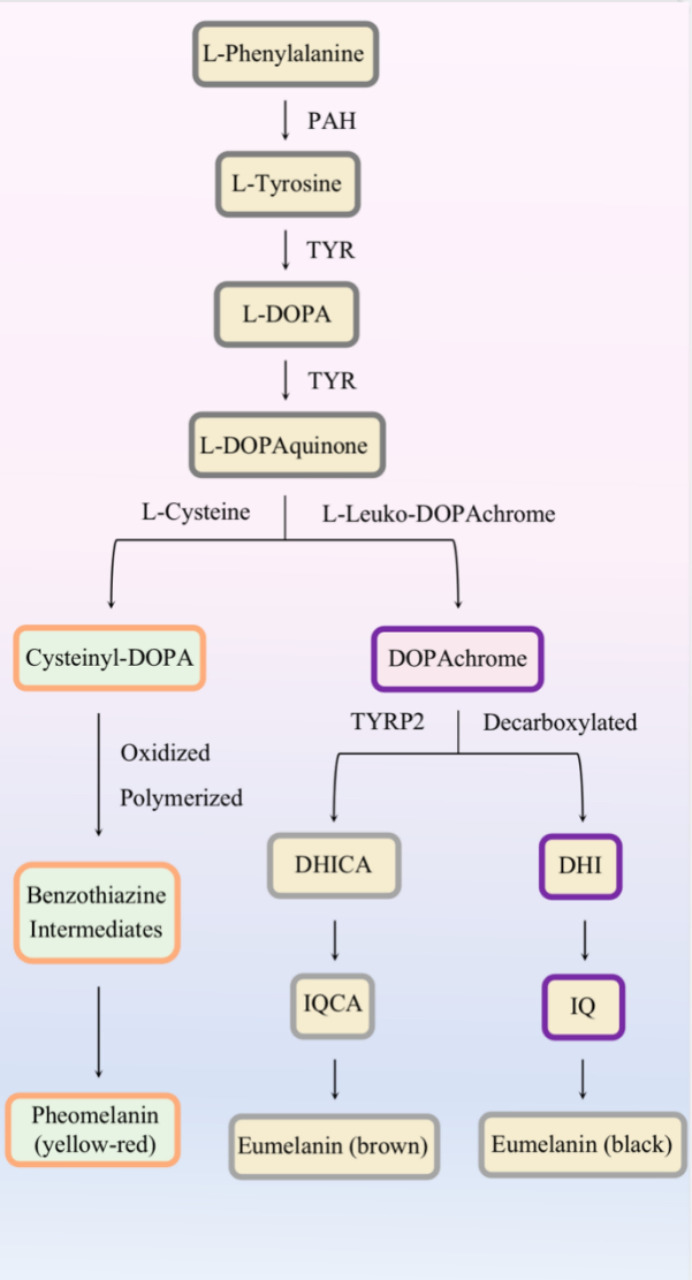
Melanin biogenesis.

Eumelanin and pheomelanin are synthesized in stages III–IV under the regulation of melanogenic enzymes (**Figure 2**). Except for the presence or absence of L-cysteine, altered pH in melanosomes can influence the balance between pheomelanogenesis and eumelanogenesis (26, 27).

The melanosomes are trafficked through a centrifugal route in the melanocytes; melanosomes are transferred from the perinuclear area to the dendrites through complementary routes mediated by microtubules (MTs) and actin filaments (AFs) (28). These trafficking mechanisms are independent: maturing melanosomes move to the periphery of melanocytes through (driven by kinesin/dynein motors) during long-distance and bidirectional transport. Once at the periphery, these pigment-deposited organelles are transferred by actin-based Rab27a/Melanophilin/Myosin-Va complex to undergo short-distance dispersion, thereby preventing reverse transport along MTs (29, 30).

The mechanism by which melanosome transfers into the keratinocytes remains elusive; however, four classic models have been proposed to explain this process: 1) exocytosis, 2) cytophagocytosis, 3) fusion of plasma membrane, and 4) membrane vesicle. Among them, exocytosis may be the most plausible mechanism, as the melanocytes in the extracellular space and melanin in keratinocytes are only surrounded by a single membrane lacking TYRP1. After entering the keratinocytes, the microtubules, actin cytoskeleton, centrosomes, and centriolar satellites likely facilitate melanin granule distribution in keratinocytes (16).

### 2.2 Melanogenesis regulation

Intrinsic and extrinsic factors regulate melanogenesis by various signal pathways, including protein kinase A (PKA), mitogen-activated protein kinase (MAPK), protein kinase C (PKC), WNT/β-catenin, and bone morphogenetic protein (BMP)/Smad cascades. Microphthalmia-associated transcription factor (MITF) in its phosphorylated active form plays a vital role in regulating these cascades; it enhances the expression of melanogenic enzymes, Rab27a protein, and the melanosomal matrix protein PMEL17 (31).

#### 2.2.1 PKA cascade

The activation of adenylate cyclase (AC) catalyzes the conversion of abundant ATP into the second messenger cyclic AMP (cAMP), which attaches to the R-subunit of PKA, thereby activating PKA. PKA subsequently phosphorylates the cAMP response element-binding protein (CREB) and salt-inducible kinase (SIK). CREB can directly enhance the *MITF* overexpression (32). SIK, which is suppressed after phosphorylation, releases more unphosphorylated CREB-regulated transcription coactivator (CRCT), which shuttles into the nucleus and binds to the already activated CREB. This complex cooperatively activates the promotor of MITF (33–35).

Alpha-melanocyte-stimulating hormone (α-MSH) and catecholamines can regulate melanogenesis *via* the cAMP-PKA pathway. Impairment of the keratinocyte DNA results in the upregulation of *p53* and, subsequently, the pro-opiomelanocortin (*POMC*) gene (36). Proteolytic cleavage of the POMC protein results in the formation of adrenocorticotropic hormone (ACTH) and α-MSH. α-MSHs produced from keratinocytes act as agonists to melanocortin 1 receptor (MC1R) on melanocytes, thereby increasing cAMP levels. Epinephrine and norepinephrine, which are catecholamines, act on their G protein-coupled receptors (GPCRs); the binding of these two first messengers to GPCRs separates Gα_s_ subunit and stimulates the production of AC (32).

#### 2.2.2 MAPK cascade

MAPK signaling is achieved through the following process: mitogen-activated protein kinase kinase kinase (Raf or MAPKKK) activates mitogen-activated protein kinase kinase (MEK or MAPKK) and, consequently, extracellular signal-regulated kinase (ERK or MAPK). The activation of downstream Raf-MEK-ERK boosts the transcription of *CREB* and *MITF* (37).

Stem cell factor (SCF) (38), basic fibroblast growth factor (bFGF) (39), and hepatocyte growth factor (HGF) (40) can bind to their tyrosine kinase receptors, c-Kit, bFGFR, and c-MET, respectively. Once bound, these receptors dimerize to boost the activity of tyrosine kinases in the intracellular juxtamembrane region to control autophosphorylation. Phosphorylated tyrosine residues conscript Src homology 2 (SH2) and pTyr-binding (PTB) domains (41). This alteration converts Ras GDP into Ras GTP-binding proteins, which are essential for the activation of Raf-1, which consequently activates the MAPK cascade.

#### 2.2.3 PKC cascade

The PKC pathway, which regulates melanogenesis, can be induced by the binding of endothelin 1 (EDN1) to its GPCR. After the complex formation, the G_αq_ unit activates phospholipase C_β_ (PLC_β_), which hydrolyzes phosphatidylinositol 4,5-bisphosphate (PIP2) to inositol triphosphate (IP3) and diacylglycerol (DAG). IP3 elicits a strong cytosolic Ca^2+^ response in melanocyte dendrites (42, 43), whereas DAG activates PKC, which can enhance the expression of MITF directly or indirectly *via* the MAPK cascade (44).

#### 2.2.4 WNT/β-catenin cascade

Frizzled receptors (FZD) bind to the transmembrane molecule LRP5/6 to form the LRP-FZD dimer complex that modulates cell differentiation and proliferation (45). As a ligand of the LRP-FZD, wingless-type MMTV integration site family members (Wnt), whose expression can be upregulated by exposure to a high dose of UV rays (46). This excessively expressed growth factor phosphorylates and inactivates glycogen synthase kinase 3β (GSK3β) after binding to the LRP-FZD complex. In the absence of active GSK3β, the accumulated β‐catenin protein in the cytoplasm is then translocated into the nucleus, where it interacts with T-cell Factor (TCF)/Lymphoid Enhancing Factor 1 (LEF1) to increase MITF levels (47, 48).

#### 2.2.5 BMP/Smad cascade

BMP regulates dorsoventral and anterior/posterior axis formation, particularly in the neural crest cells, which require the expression of BMP2 and BMP4 (49). Signals generated by the assembly of BMP-BMPR and SMAD1/5/8 are recruited in the cytoplasm to be phosphorylated and to form a complex with co-SMAD4. This complex subsequently relocates into the nucleus to regulate MITF expression (50).

## 3 Postinflammatory hyperpigmentation

PIH is an acquired pigmentary disorder that primarily affects patients with darker skin types (Fitzpatrick types III–VI). Nonetheless, all skin types suffer from PIH owing to endogenous and exogenous injuries. Several experiments exploring inflammatory mediators have been conducted based on the specific mechanism of inflammatory responses (**Table 1**).

**Table 1:** The influence of inflammatory cytokines on melanogenesis and its mechanisms.

Cytokines	Main source	Impact on melanogenesis	Mechanisms	Refs.
IL-1α	Langerhans cells/Keratinocytes	Promotion	Pigment enhanced when binding with KGF	(51)
IL-1β	Macrophages/Keratinocytes	Demotion	NF-κB, JNK pathways	(52)
IL-4	Th2 cells/Basophils	Demotion	JAK2-STAT6 pathways	(53)
IL-6	Keratinocytes/Fibroblasts	Demotion	MITF, TYR, NHM viability	(54)
IL-13	Th2 cells	Demotion	JAK2-STAT6 pathways	(55, 56)
IL-17	Th17 cells	Demotion	MAPK, PKA pathways	(57, 58)
IL-18	Macrophages/Keratinocytes	Promotion	MAPK, PKA pathways	(59)
IL-33	Keratinocytes/Fibroblasts	Promotion	MAPK, PKA pathways	(60–63)
PGE2	Keratinocytes/Melanocytes	Promotion/Demotion	MAPK, PKA pathways, Formatting melanocyte dendrites	(64, 65)
IFN-γ	T cells	Demotion	JAK1-STAT1 pathways, Melanocytes apoptosis, Melanosomes mature and convey, Metabolite of tryptophan	(59, 66–71)
TNF	Macrophages/Keratinocytes/Th1, Th17, and Th22	Demotion	MAPK, PKA pathways	(58, 72, 73)
GM-CSF	Keratinocyte	Promotion	Melanocyte proliferation	(74)

KGF, keratinocyte growth factor; NF-κB, nuclear factor kappa-B; JNK, Jun N-terminal kinase; MITF, microphthalmia-associated transcription factor; TYR, Tyrosine; NHM, normal human melanocyte; PKA, protein kinase A; ERK or MAPK, extracellular signal regulated kinase; JAK, The Janus kinase; STAT, signal transducer and activator of transcription.

The advent of genomic medicine has confirmed the indispensable role of PKA and MAPK signaling pathways in various biological processes, especially those related to oncogenesis or its progression. Similarly, most inflammatory cytokines regulate melanogenesis *via* these two cascades. Interleukin (IL)-18 and IL-33 contribute to the expression of MITF and other related enzymatic expressions by stimulating the PKA and MAPK pathways (59, 60). IL-33 expression, influenced by IL-17 and interferon (IFN)-γ, establishes a negative feedback loop resulting in pigmentation (61, 62). However, IL-33 is also speculated to have a positive feedback loop with tumor necrosis factor alpha (TNF-α), which induces melanocyte death resulting in vitiligo (63).

IL-17 adversely affects melanogenesis because the expression of *MITF* and its downstream genes increases on blocking with anti-IL-17RA (57). IL-17 and TNF synergistically inhibit melanin production (58). Neutralization of TNF and IL-17 with monoclonal antibodies (mAbs) increased the levels of c-KIT, MITF, and TYRP2 (58, 72). This suggests that TNF induces IL-17 to exert a negative effect on melanin synthesis through MAPK and PKA signaling pathways (72, 73).

Under the control of phospholipase A2 (PLA2), cyclooxygenase (COX), and prostaglandin E synthase (PGES), PGE2 is released by keratinocytes and epidermal melanocytes (64). In response to PGE2, its receptor EP3 suppresses cAMP production, thereby preventing pigmentation. In contrast, EP4 receptor activation may increase basal cAMP levels, stimulating tyrosinase and the formation of dendrites in melanocytes (65).

Janus kinase (JAK) and signal transducer and activator of transcription (STAT) have been known as rapid membrane-to-nucleus signaling modules. They have been associated with cancer and inflammation for the past two decades. However, compared with PKA and MAPK pathways, the regulation of melanogenesis by the JAK-STAT pathway is relatively unknown. However, several cell cytokines have been reported to alter melanocyte function through the JAK-STAT pathway.

Studies on normal human melanocyte (NHM) cultures confirmed that IL-4 produces melanin by downregulating MITF and dopachrome tautomerase expression through the JAK2-STAT6 signaling pathway (53). IL-13, which shares JAK2-STAT6 signaling pathways with IL-4 (55), can inhibit the mRNA and protein expression levels of both tyrosinase and Dopachrome Tautomerase (DCT), thus impacting melanin synthesis (56).

By upgrading the phosphorylation of the JAK1-STAT1 cascade, IFN-γ mediates reversible and independent MITF dyspigmentation through Recombinant Interferon Regulatory Factor 1 (IRF1) binding and DCT promoter repression in a dose-independent manner (66). By associating CREB -binding protein (CBP) with elevated STAT1, IFN-γ can also inhibit the binding between CBP and CREB. In this manner, by not affecting CREB phosphorylation, α-MSH-induced melanogenesis exhibits inhibition (67).

IFN-γ can induce hypopigmentation *via* other mechanisms, including apoptosis in melanocytes (68), arresting melanosome maturation and transportation (66), and metabolism of tryptophan (69–71). Moreover, it is also known that IFN-γ shares crosstalk with IL-18, wherein IFN-γ inhibits IL-18-induced melanogenesis indirectly (59).

Other than the cascades referred to in this section, some cytokines can also influence pigmentary deposition *via* other mechanisms. IL-1α, a subtype of IL-1, initiates IL-1 receptor type I (IL-1RI), increasing little pigment deposition. However, this effect is in combination with the keratinocyte growth factor (KGF) (51). Conversely, IL-1β, another form of IL-1, is presumed to inhibit MITF through the nuclear factor kappa-B (NF-κB) and Jun N-terminal kinase (JNK) pathways (52). IL-6 treatment, analyzed *in vitro* and *in vivo*, decreases the viability of NHM, MITF, and TYR in a dose-dependent manner (54). Anti-granulocyte-macrophage colony-stimulating factor (GM-CSF) treatment led to the nulling of the keratinocyte regulates Melan-A melanocyte proliferation, indicating that the proliferation of melanocytes is augmented by GM-CSF (74).

## 4 Inflammation after burn injury

Pathogen-associated molecular patterns (PAMPs) and damage-associated molecular patterns (DAMPs) are the two main mechanisms that trigger inflammatory responses. PAMPs are caused by diverse microbial molecules, especially lipopolysaccharides (LPSs) discovered in Gram-negative bacteria. In contrast, DAMPs require environmental alterations, such as trauma, thermal stimuli, or other damage to cause sterile inflammation (75). Toll-like receptors (TLRs), members of pattern recognition receptors (PRRs), stimulated and shared by both DAMPs and PAMPs, can activate intracellular signals to regulate inflammation and ensure similarity of inflammatory responses even under different stimuli (76). The activation of TLRs releases many inflammatory cytokines from the damaged tissue into circulation through myeloid differentiation primary response protein-88 (MyD88), leading to the transcription of activator protein 1 (AP-1) and NF-κB (77). The increase in cytokines, locally and systemically, causes infection, impaired healing, pigmentation disorders, and systemic inflammatory response syndrome.

Burn injury, a unique DAMP (78), affects the skin, which is the barrier protecting the human body from pathogens and environmental stimuli. The impacted skin forms debris of tissues and eschar, which serve as a reservoir for diverse bacteria and PAMPs, especially around hair follicles with a higher bacterial load (79). Additionally, TLR expression on dendritic cells is upregulated in severe burn wounds (80).

The downstream nucleus, called cytokines in circulation, has been described for appraising and monitoring patients with burns. IL-1β has been reported for its poor outcome correlation, such as systemic inflammatory response syndrome or even death (81, 82). IL-6 specifically induces remote organ inflammation (81–89) (**Table 2**). IL-8 and IL-10 expression levels are upregulated (8, 83–89), whereas IL-4 and IL-7 levels are downregulated (84). Meanwhile, the plasma levels of other cytokines, such as granulocyte colony-stimulating factor (G-CSF), GM-CSF, macrophage inflammatory protein-1 (MIP-1), and TNF-α, were also observed to increase in patients with burn wounds (83–89). Similarly, changes in cytokine levels in local burn wounds have also been observed. In the burn trauma mouse model, levels of IL-6, TNF-α, and MCP-1 showed a significant increase (90). However, Schwacha et al. (91) reported contradictory results based on their burn wound mouse models.

**Table 2:** Inflammatory cytokine character and alteration in the burn model.

Cytokines	Character	Sample	Site	Upregulation or downregulation	Refs.
IL-1β	Pro-inflammatory factor	Human/Mouse	Plasma	Up	(81, 82)
IL-4	Anti-inflammatory factor	Human	Plasma	Down	(84)
IL-6	Pro-inflammatory factor	Human/Mouse Mouse	Plasma Wound	Up Up Down	(81–89) (90) (91)
IL-7	Pro-inflammatory factor	Human	Plasma	Down	(84)
IL-8	Pro-inflammatory factor	Human	Plasma	Up	(83–89)
IL-10	Anti-inflammatory factor	Human	Plasma	UP	(8, 83, 87–89)
G-CSF	Pro-inflammatory factor	Human	Plasma	Up	(85–87)
GM-CSF	Pro-inflammatory factor	Human	Plasma	Up	(85)
MIP-1	Pro-inflammatory factor	Human Mouse	Plasma Wound	Up Up	(83–86, 89) (90)
TNF-α	Pro-inflammatory factor	Human Mouse	Plasma Wound	Up Up Down	(87, 88) (90) (91)

## 5 Assessments and treatments

Currently, only limited assessments and treatments specifically target burn trauma-induced PIH. We have summarized and evaluated the currently available therapies for PIH to facilitate further research on developing treatments against burn injury-induced pigmentation disorders (**Table 3**).

**Table 3:** Assessments and treatments.

Assessments			
	Type	Refs.	Review
Visual assessment	Fitzpatrick scale	(92)	Easy to practice but subjective due to its interobserver variability
	Taylor scale	(93)	
	Wood’s lamp	(94, 95)	
	Skin biopsy	(96)	Limited due to its invasiveness
Optical techniques	Polarized light photography	(97)	More reliable and reproducible but require professional training
	Tristimulus colorimetry	(98)	
	Diffuse reflectance spectroscopy	(99)	
	Hyperspectral imaging	(100, 101)	
	Reflectance confocal microscopy	(102–104)	
Treatments			
	Type	Refs	Review
Oral treatments	Tranexamic acid	(105, 106)	Curing effect is finite
	Glutathione	(107)	
	Botanical agents	(108, 109)	
Topical treatments	Hydroquinone	(110–113)	The optimal choices being more and less toxic
	Retinoids	(114, 115)	
	Azelaic acid	(116)	
	Kojic acid	(117)	
	Niacinamide	(118, 119)	
	Traditional Chinese medicines	(120)	
Procedural treatments	Chemical peeling	(121–123)	Risks of worsening PIH due to skin irritability
	Laser technology	(124)	

### 5.1 Visual assessment

The use and application of the visual hyperpigmentation scale in clinical practice are straightforward. The Fitzpatrick scale was created to classify human skin into six phototypes (I–VI) based on tanning response to UVR (92). The Taylor hyperpigmentation scale with 150 gradations for hyperpigmentation was proposed to obtain further information; this scale comprises plastic cards in 15 different colors (A–J) and 10 pigmentary gradations for each color card (93).

Wood’s lamp (340–400 nm, with maximum output at 365 nm) emits UV and visible light (94). Hence, epidermal PIH appears darker in contrast to unaffected healthy skin. However, PIH that occurs in the dermis cannot be distinguished as well as that in the epidermis under the Wood’s lamp examination. The accuracy of Wood’s lamps in differentiating between dermal and mixed melasma can be improved with the assistance of dermoscopy (95).

Skin biopsy is a standard method to support PIH diagnosis, excluding certain hyperpigmentation disorders such as melasma and drug-induced hyperpigmentation. Histopathologically, PIH presents perivascular or perifollicular lymphocytic inflammation, dermal melanophages, and epidermal melanin without basal cell vacuolization (96). Using skin biopsy as a criterion for diagnosing PIH and other similar disorders is limited owing to its invasive nature.

### 5.2 Optical techniques

Unlike subjective clinical assessments, noninvasive technologies supplement more credible and repeatable outcomes for PIH. These consist of polarized light photography, colorimetry, diffuse reflectance spectroscopy (DRS), hyperspectral imaging (HSI), and reflectance confocal microscopy (RCM).

Polarized light photography is sensitive to detecting dermal changes, especially vascular changes. When using parallel polarizing filters, melanosis on the skin surface can be visualized using the incident light source at a certain angle to the camera. Cross-polarized photography can be used to visualize hyperpigmentation and subsurface features such as vascularity, whereas parallel-polarized photography is used for skin texture (97).

Tristimulus colorimetry is analogous to human eyes in perceiving color. There are three axes, including L* (lightness-darkness) axis, a* (red-green) axis, and b* (blue-yellow) axis, which describe and plot color in a three-dimensional space. L* and b* values are used to measure pigmentation (98).

DRS, an *in vivo* measurement, can compute and quantify the biochemical concentrations of skin melanin, oxyhemoglobin, and deoxyhemoglobin, according to their absorbance characteristics (99). It is also used to estimate PIH because of its function of quantifying melanin.

HSI measures spectral bands and provides sensitive measurements. In reflectance spectra, it can capture subtle alterations and quantify optical properties of the skin, containing information about hemoglobin and melanin (100). Recently, this method was integrated with machine learning—an established structure-adaptive normalized convolution algorithm (101).

RCM, a great advance in dermatology, provides high-contrast images of melanin granules. By emitting and detecting near-infrared wavelength laser beam, RCM can visualize the layers between the epidermis to the upper reticular dermis to a depth of 250–300 μm (102). Owing to the relative differences in refractive indices and sizes of cell organelles and keratin, signal contrast of melanin can be obtained (103). Therefore, RCM has been used in evaluating the PIH model dynamically (104).

### 5.3 Oral treatments

Tranexamic acid (TA), widely used to fix abnormal fibrinolysis, effectively inhibits melanogenesis. TA likely blocks UV-induced plasmin activity, which decreases the levels of raw material of tyrosinase— arachidonic acid and prostaglandins (105). Oral TA effectively decreases the mean melanin index in TA-treated keratinocyte-conditioned medium (KCM) (106).

Glutathione, an antioxidant with skin-lightening activity, can switch eumelanin production into pheomelanin. The reaction of the glutathione thiol with dopaquinone forms a sulfhydryl–dopa conjugate that results in the formation of pheomelanin instead of eumelanin (107).

Other botanical agents proposed for their lightening effects include ginseng and grape seed. Ginsenosides, the major active compounds of ginseng, have a synergistic effect on bFGF-induced antiproliferation of melanocytes *via* ERK cascades (108). Proanthocyanidins extracted from grape seeds can potentially regulate the NHM cell cycle and inhibit the production of melanogenic enzymes (109).

### 5.4 Topical treatments

Hydroquinone, the most common ingredient of skin-lightening agents, can prevent the conversion of Dihydroxyphenylalanine (DOPA) to melanin and restrict the differentiation of melanocytes from neural crest cells (110). Hydroquinone is the most effective in the concentration of 2%–5%. To guarantee its efficiency and minimize its defects, many combination formulae have been approved by the US Food and Drug Administration (FDA) (111). However, hydroquinone topical creams reportedly cause skin toxicity and other side effects (112). Therefore, mequinol (4-hydroxyanisole) may be used as a substitute for the parent compound to reduce skin irritation (113).

Retinoids, which are vitamin A analogs, facilitate epidermal turnover, thus removing melanin. Isotretinoin, adapalene, and tazarotene are three mainstream retinoids that have been proven to be useful in treating hyperpigmentation (114). Although its irritant activity is not as high as the other two forms, isotretinoin should be prescribed in high concentrations (115).

Azelaic acid (AzA) is used as a depigmenting drug in acne owing to its anti-inflammatory, antibacterial, and antioxidant properties. In acne-related PIH, alleviation and clearance of pigmentation were observed at the end of the study (116). In addition, AzA is safer and was assigned pregnancy category B by the FDA for its mild side effects.

Kojic acid (KA) inactivates tyrosinase by chelating copper, which is the prosthetic group for this enzyme; it is effective in concentrations ranging from 1% to 4% (117). The maximum potential human systemic exposure dose (SED) of KA is 0.028 mg/kg/day. KA reduces the synthesis of melanin and is less toxic to melanocytes (117).

Niacinamide, an amide form of vitamin B3, was observed to impede the transfer of melanin to epidermal keratinocytes in a keratinocyte–melanocyte cocultured system (118). The level of nicotinamide nucleotide transhydrogenase (NNT) is low in individuals with PIH (119). Thus, the effect of nicotinamide on NNT activity and skin pigmentation alteration can be explored in the future.

Many traditional Chinese medicines (TCMs) with skin-whitening functions that are recorded in prescriptions, such as Fructus Ligustri Lucidi, *Hedysarum multijugum* Maxim., *Ampelopsis japonica*, Pseudobulbus Cremastrae seu Pleiones, and Paeoniae Radix Alba, have also been observed to inhibit TYR expression and activity (120).

### 5.5 Procedural treatment

Chemical peeling, which includes the removal of the superficial and deeper skin layers to regenerate the epidermis and part of the dermis, causes reversible damage to the skin and redistributes melanin. The superficial peeling strips the stratum corneum, whereas medium to deeper peelings penetrate the papillary and reticular dermis. Superficial peeling agents contain glycolic acid (GA), salicylic acid (SA), trichloroacetic acid (TCA), and tretinoin. In a study, repeated use of 5% GA improved brightness and reduced redness with respect to the melanin and erythema in the fourth week (121). SA modifies skin indices, such as melanin, pores, and texture; this alteration is also enhanced by oral administration of isotretinoin (122). TCA peels improve signs of photoaging such as hyperpigmentation, erythema, and fine lines with repeated treatment (123). Therefore, superficial peeling is the most viable treatment for removing melanin. In contrast, deeper peeling agents may result in PIH owing to irritation.

According to the principles of photothermolysis, current laser technologies selectively and specifically destroy targeted tissue. Modern melanin-directing laser machines consist of a Q-switched (QS) laser and a picosecond (PS) laser. The wavelengths used in these two systems are Nd : YAG (532 nm), Nd : YAG (1,064 nm), and alexandrite (755 nm) (124). All these new generations of lasers reflect patient selection with safer technologies and less postoperative recovery time. However, although it is anticipatory and largely transient, care and attention must be provided to avoid laser irradiation-induced PIH.

## 6 Discussion

In the field of burns, more attention has been paid to the patient’s systemic status and the maintenance of all vital organs, and little research has been done on changes in skin color after burns. Although not well documented, it is clinically possible to observe that some burn patients with a severe inflammatory response have a greater proportion of hyperpigmentation than less severe patients. Therefore, this suggests a possible relationship between burns, inflammation, and melanogenesis. Few scholars have focused on this phenomenon, and there is no definitive evidence for the relationship between these three. Therefore, to fill this research gap, this review discusses the relationship between the three as much as possible in relation to the existing literature.

It summarizes pigment formation and its transport, regulation of melanogenesis, mechanisms of PIH, and changes in inflammatory cytokines induced by burns. It bridges the gap between PIH and burn wounds, thereby providing insights that would aid in its treatment and management. Pigmentation is mainly regulated through various pathways, which, in turn, are regulated by various inflammatory factors, resulting in PIH. Burns, as a stimulus to DAMP, often cause cytokine storms. Burn wounds often appear hyperpigmented. This suggests that the pigmentary changes in burn wounds may be caused by changes in the levels of inflammatory mediators. Collectively, controlling inflammation in burn wounds may help reduce hyperpigmentation.

The mechanism of PIH in the current study mainly focuses on the PKA and MAPK pathways. We possess limited information regarding the other mechanisms of pigmentation, such as JAK-STAT, and the interaction among the various pathways. In addition, most PIH models are based on vitiligo and acne, with few studies on burn models. Changes in inflammatory mediators after burns are mainly observed in the plasma, with few studies focusing on their profiles in wounds. Therefore, much effort should be paid to developing effective burn models for studying PIH and to excavating more unknown mechanisms, especially pathways of PIH, and how these mechanisms promote or inhibit it.

Few assessments and therapies exist for PIH, specifically those induced by burns, and these mainstream approaches treating PIH are mainly directed toward melanogenesis as a phenotype to address hyperpigmentation. Their mechanisms for reducing melanogenesis are not solely, e.g., anti-inflammatory, antioxidant properties, direct reduction of melanocytes, accelerated exfoliation of the stratum corneum, etc. Some treatments, such as systemic and topical drugs and laser therapy, have been used to relieve pigmentation disorders in regular clinical practice. Procedural treatments might worsen PIH. Despite their toxicity, topical treatments are the current optimal choices owing to the limited number of oral drugs to cure PIH.

Since the inflammatory phase partially overlaps the pigmentation phase, further research is required to shed light on the alterations in inflammatory cytokines in the burn wound site, especially in humans. Additionally, the direct or indirect action of these alterations on the pigmentation must be elucidated. If the direct action is confirmed, anti-inflammatory and pigment-inhibiting drugs and treatments would be potential and superior choices for alleviating PIH in patients suffering from burn wounds. Finally, persistent effort is required to identify nontoxic and reliable whitening agents.

## Author contributions

CZ collated the topics for review, retrieved the relevant literature, and wrote this review. DX, GL, PL, KS, FL, and JZ edited this review. All authors contributed to the article and approved the submitted version.
